# Annotations

**Published:** 1906-04-28

**Authors:** 


### ANNOTATIONS.

### The Dangers of Food Preservatives.

Diseases and dangers to our bodily health generally come upon us unawares. They may frequently be said to stab us in the dark, and, perhaps, never so stealthily as when they strike us under the cover of the food which we take to nourish and strengthen our bodies. When such a blow falls it may be terrible in its severity. The healthy man may be deprived of life within twenty-four hours. Rapidly fatal results of this character are, however, rarely, if ever, directly a consequence of the use of food preservatives. Subtle microbes are generally the offending agents. Yet it is possible that food preservatives may aid these microbes to obtain entrance to the body. In such an article as milk, the preservative may restrain the growth of micro-organisms which will affect the taste of the milk yet not materially inhibit the increase of other more dangerous germs. Where the taste is not affected there is nothing to warn the drinker that he is swallowing poison. The *Sanitary Record* is therefore no doubt wise when it expresses the opinion that milk "should contain no preservative whatever." While, however, the greatest dangers which may be hidden in food are due to the presence of micro-organisms, there are other lesser dangers which may follow the frequent entrance into the body of small doses of some drug used as a preservative. Some people are much more liable to such dangers than others. "What is one man's food is another man's poison" is very true of drugs. Just as certain persons are easily upset by a small quantity of some article of diet, so are some people curiously susceptible to very small doses of some drugs, and those doses may lead to troublesome affections, which are intractable so long as the cause remains unsuspected. Drugs used as preservatives are, therefore, a source of danger. It may appear to be the dictum of a faddist to say that they should never be used. It is, however, difficult to draw the line in such matters. Use so often degenerates in abuse that the simplest course—complete exclusion—is the safest.

### A Plea for the Endowment of Research in the Field of Insanity.

It is to be hoped that the excellent articles recently contributed to the columns of the *Times* under the title "The Growth of Insanity" will assist in the dissipation of the *non possumus* attitude which has in the past done so much to paralyse research into the causation of the insanities. So long as public opinion is content to say, "Insanity is an extra-corporeal affliction; we must protect its victims, but cannot expect to fathom its causation," so long will our knowledge of insanity mark time, while that of admittedly corporeal afflictions continues to make immense strides. As is pointed out by the writer in the *Times*, this attitude on the part of the lay world—which supplies the directorate of our asylums—has led to an arrangement of the medical offices in mental institutions which is fatal to the prosecution of research; and it is by research, not by vague academic argument, that progress is to be achieved. Under the existing régime the superintendent of an asylum is indeed a qualified practitioner, but in fact he is quite as much occupied with administrative detail as he is with the true duties of an alienist. He is the chief executive officer of a small town, one might say, and has no time, even if he has the inclination and capacity, for complex scientific researches. In consequence the medical care of the inmates devolves upon the junior medical officers of the institution, and these seem to be content, and perhaps with good reason, to confine their attentions to the obviously bodily maladies of their patients. The problem which faces the country may be stated thus: The bulk of informed opinion credits insanities with an organic basis. The successful investigation of this organic basis postulates research of a high order, for the problems presenting themselves are very intricate. Therefore the most urgent necessity is the endowment of research in this direction, and, more, an endowment sufficiently liberal to attract the high intellectual faculties which the difficulties of the task demand.

### "The Paris Medical Journal."

This, the latest enterprise in medical journalism, is an attempt to present a monthly review of French medicine for the benefit of the English-speaking peoples. In a brightly-written "prefatory note" the editors announce their intention to provide, month by month, a summary of matters medical, both Parisian and provincial; they also propose to review the more important communications made to the Academy of Medicine and to the various medical and surgical societies; and each number of the journal is to contain a description of one of the Paris hospitals and a description of a French health resort. The first issue promises well for the liberal redemption of this attractive programme. It gives us an account of the career of Professor Debove, who is much more than a name to the majority of our readers. There is also an illustrated description of the history of the Hôtel-Dieu, and a useful map showing the various French watering places and other health stations. All these are in addition to the main substance of the journal, which comprises a series of well-arranged digests from the several Paris cliniques, summaries of contributions to medical journals, and papers read before learned societies. There are also some useful editorial notes, a list of the various post-graduation courses, and items about hospitals and medical societies that cannot fail to be useful to medical visitors to Paris. The new venture is, we think, a happy inspiration, and one likely to be of service both to the progress of medical science and to the best interests of the peoples between whom it will form a means of communication. We have nothing but praise for the first issue, in which the articles are well selected, well arranged, and well written. The existence of a sound and responsible journalistic instinct is readily perceived by the experienced eye, and unless we are much in error our new contemporary is at the beginning of a useful and successful career. The editors are Dr. A. A. Warden and Dr. Edmund L. Gros.

